# Induced Hydrophilicity and In Vitro Preliminary Osteoblast Response of Polyvinylidene Fluoride (PVDF) Coatings Obtained via MAPLE Deposition and Subsequent Thermal Treatment

**DOI:** 10.3390/molecules25030582

**Published:** 2020-01-29

**Authors:** Luminita Nicoleta Dumitrescu, Patricia Neacsu, Madalina G. Necula, Anca Bonciu, Valentina Marascu, Anisoara Cimpean, Antoniu Moldovan, Andrei Rotaru, Valentina Dinca, Maria Dinescu

**Affiliations:** 1 INFLPR-National Institute for Laser, Plasma and Radiation Physics, Bvd. Atomistilor, Nr. 409, Magurele (Ilfov), 077125 Bucharest, Romania; nicoleta.dumitrescu@inflpr.ro (L.N.D.); anca.bonciu@inflpr.ro (A.B.); valentina.marascu@inflpr.ro (V.M.); antoniu.moldovan@inflpr.ro (A.M.); 2 Faculty of Physics, University of Bucharest, RO-077125 Magurele, Romania; 3 Department of Biochemistry and Molecular Biology, Faculty of Biology, University of Bucharest, 91-95 Splaiul Independentei, 050095 Bucharest, Romania; neacsu.patricia88@gmail.com (P.N.); necula.madalina92@gmail.com (M.G.N.); anisoara.cimpean@bio.unibuc.ro (A.C.); 4 Department of Biology and Environmental Engineering, Faculty of Horticulture, University of Craiova, Str. A.I. Cuza, Nr. 13, 200585 Craiova, Romania; 5 Department of Chemical Thermodynamics, Institute of Physical Chemistry “Ilie Murgulescu”, Romanian Academy, Str. Splaiul Independentei, Nr. 202, 077125 Bucharest, Romania

**Keywords:** PVDF, MAPLE, coatings, induced-hydrophilicity, polymeric biointerface, thermal treatment

## Abstract

Recent advancements in biomedicine have focused on designing novel and stable interfaces that can drive a specific cellular response toward the requirements of medical devices or implants. Among these, in recent years, electroactive polymers (i.e., polyvinylidene fluoride or PVDF) have caught the attention within the biomedical applications sector, due to their insolubility, stability in biological media, in vitro and in vivo non-toxicity, or even piezoelectric properties. However, the main disadvantage of PVDF-based bio-interfaces is related to the absence of the functional groups on the fluoropolymer and their hydrophobic character leading to a deficiency of cell adhesion and proliferation. This work was aimed at obtaining hydrophilic functional PVDF polymer coatings by using, for the first time, the one-step, matrix-assisted pulsed evaporation (MAPLE) method, testing the need of a post-deposition thermal treatment and analyzing their preliminary capacity to support MC3T3-E1 pre-osteoblast cell survival. As osteoblast cells are known to prefer rough surfaces, MAPLE deposition parameters were studied for obtaining coatings with roughness of tens to hundreds of nm, while maintaining the chemical properties similar to those of the pristine material. The in vitro studies indicated that all surfaces supported the survival of viable osteoblasts with active metabolisms, similar to the “control” sample, with no major differences regarding the thermally treated materials; this eliminates the need to use a secondary step for obtaining hydrophilic PVDF coatings. The physical-chemical characteristics of the thin films, along with the in vitro analyses, suggest that MAPLE is an adequate technique for fabricating PVDF thin films for further bio-applications.

## 1. Introduction

Novel, advanced, synthetic polymeric biomaterials have attracted attention in recent decades due to their processability, morphological characteristics, surface chemistry, and wide range of potential applications in the sector of electronic devices and for biomedical science (i.e., sensors, biosensors, membranes, etc.) [1,2,3]. Among these materials, polyvinylidene fluoride (PVDF) is a semi-crystalline fluoropolymer presenting a number of characteristics that make it a versatile biomaterial: insolubility, low processing temperature, chemical resistance, stability in biological media, in vitro and in vivo nontoxicity [4,5,6,7], piezoelectricity, and pyroelectricity [8,9]. This implies the possibility for it to be used as multifunctional bio-coating. Depending on the desired bio-application, specific requirements for the surface characteristics and microarchitecture need to be considered in correlation with the biomolecules or cell types used for in vitro studies [4]. Therefore, it is essential that the mechanical, physical, and chemical characteristics, as well as the hydrophilic or hydrophobic nature of the substrate, are well-known, since they will influence the means of interaction between cells and surfaces [1,4,5,6,7]. In this context, a study conducted by Szewczyk et al. evaluated the cellular response to a smooth PVDF film compared with biomimetic, highly porous, and rough surfaces [10]. To obtain this film, no chemical modifications were applied, and the geometry of the surfaces was designed only through the innovative combination of solvents, including dimethylacetamide and acetone, and by controlling humidity and temperature in the films’ processing. The results obtained showed that the rough PVDF film enhanced the osteoblast adhesion by over 44% compared with the smooth film. At the same time, another study evaluated the biological effect generated by a PVDF nanofibrous scaffold compared with drop-cast membranes [11]. Moreover, an oxygen plasma treatment was performed in order to obtain hydrophilic PVDF surfaces. The results showed that osteoblast cells cultured on hydrophilic PVDF scaffolds displayed better cell spreading and adhesion over the non-treated ones, as well as an enhanced integration into the scaffold. Additionally, the drop-cast PVDF exhibited a lower degree of piezoelectricity comparing to the electrospun equivalent, which was quantified by measuring the intracellular calcium influx. Furthermore, the case of PVDF when the absence of the functional groups on the fluoropolymer, due to low free surface energy, tends to conduct to a deficiency in the cell adhesion and proliferation is well-known [12]. Therefore, considerable efforts have been made for improving the surface hydrophilicity of PVDF polymer by altering the surface chemical composition using various methods: blending the polymer with chemical modifiers [13], plasma treatment, reactive ion etching (RIE) [14,15], etc. Nevertheless, depending on the application, there usually exists the need for PVDF processed in different shapes. Bulk/pure PVDF and copolymer scaffolds [16], nanofibers [17], membranes [3], and coatings have been produced for a wide range of applications: sensors [1], microelectronics [18], optoelectronics [19], biosensors [2], and bio-medical technology [4]. To obtain the abovementioned structures, methods such as spin coating [8], electrospinning electrospray [16,17], immersion precipitations [20], and pulsed laser deposition (PLD) [21] have been employed. However, each of these methods has some drawbacks, since they can lead to a relatively poor adherence of PVDF to the underlying substrate, due to its low surface energy [13]. For example, spin coating, despite being one of the most commonly used methods, does not allow the deposition of structurally complex materials or coating on any substrate, requiring in some cases intermediate adhesion layers to adapt to the coating to the substrate, limiting the efficiency of a certain application (transducer) [8,22]. The electrospinning-electrospray technique presents certain disadvantages for bio applications, such as low seeding efficiency and poor cell infiltration, in all its grades of thickness [23], as well as difficulties precisely controlling the thin film morphology when the immersion precipitation technique is used [4,24]. Nevertheless, other methods have been developed for biomedical applications when improvements in the capacity of PVDF thin films to support cell (e.g., osteoblast) adhesion and proliferation have been required (e.g., graft copolymerization or Chemical Vapour Deposition CVD polymerization [25], melt spun PVDF fiber [26], and dynamic piezoelectric stimulation [27]).

Because many materials used in surface engineering lack reactive groups for the covalent attachment, hydrophilisation through physical surface modification (e.g., coating with a hydrophilic polymer layer) or chemical treatment (e.g., plasma grafting of polar groups) are used [12]. For these materials, the major challenge of surface engineering is the introduction of reactive groups to the surface [25], the lack of control in the deposited area homogeneity, tailoring the final thickness, expense, and the process being time-consuming. In recent decades, laser techniques, especially Matrix-Assisted Pulsed Laser Evaporation (MAPLE) [28,29,30,31], have proved to be viable methods for organic material processing in tissue engineering applications [32,33,34]. This technique offers unique advantages compared to conventional deposition methods of soft materials—for example, the spin coating technique [8,22]. The MAPLE method “gently” allows the material of interest to be transferred to the substrate with little or no damage to its structure, chemical properties, and functionality; the matrix responsible for the dilution and homogenization of the material of interest absorbs the wavelength of the laser beam, is evaporated, and is then pumped out of the deposition chamber [29,30,31]. Other advantages of this technique are the low consumption of materials, the control over thickness (by monitoring the deposition rate), control over surface roughness (by changing the target characteristics and number of pulses), and uniform transfer on non-planar substrates, as well the deposition of composite or multilayer films/coatings [33,34,35,36,37]. MAPLE allows the transfer of a wide range of materials, being thus successfully used for transferring different types of organic materials and polymers, such as the chemoselective polymers [38], nanoparticles [39], electro-conductive polymers [40,41], biodegradable polymers [42], thermoresponsive polymers [43], and even active proteins [44,45], as well as layers of organic materials with graded composition [39], liquid crystals [46,47], and much other more [44,48,49].

Within this context, and given the high potential of PVDF coatings to be further used as a multifunctional and highly stable bio-interface, the main purpose of the present study was to exploit the advantages of MAPLE for the deposition of functional PVDF coatings with hydrophilic surface characteristics for the first time, as well as studying the initial pre-osteoblast response to these surfaces. As dimethyl sulfoxide (DMSO) is toxic for cells, the residual presence of this solvent within the coatings is investigated before and after the thermal treatments [49] that target the removal of DMSO, studying the effect on the PVDF layers, while the influence of DMSO on the cells are also presented.

## 2. Results and Discussions

Thin films of polyvinylidene fluoride (PVDF) for bio-applications were fabricated by means of the matrix-assisted pulsed laser evaporation (MAPLE) technique, in order to support the survival of viable osteoblasts with active metabolism, and thus be able to reverse the hydrophobic character into a hydrophilic one at the nanoscale. The obtained materials were subjected to thermal treatment (TT) at 70 °C for 5 h, in order to totally eliminate the solvent used during the MAPLE process that may adhere to the layers, and also to enhance the surface morphology for a potentially better accommodation of cells.

### 2.1. Physical and Chemical Characterization of the Polyvinylidene Fluoride Coatings

**Figure 1:**
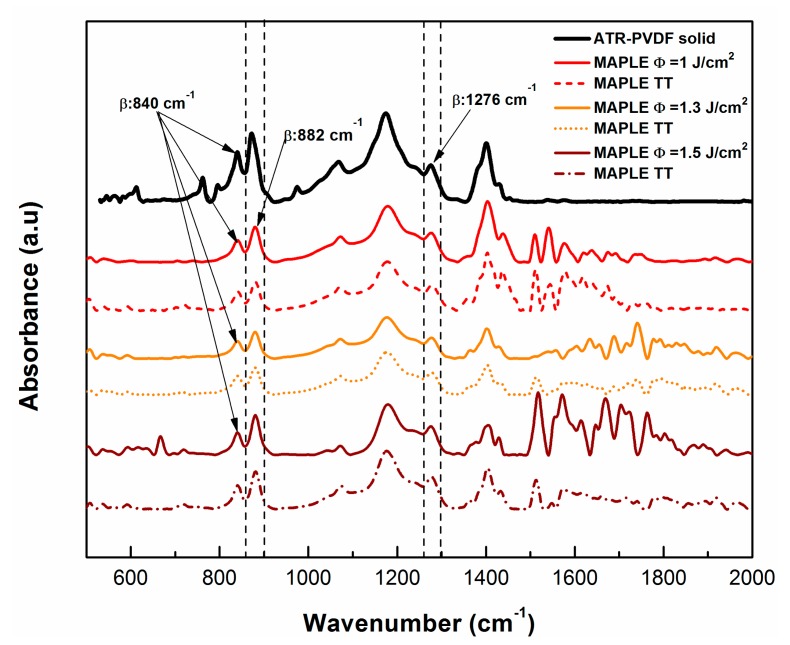
FTIR attenuated total reflectance (ATR) and FTIR spectra of PVDF coatings deposited by matrix-assisted pulsed evaporation (MAPLE) and of the coatings subjected to the thermal treatment (MAPLE TT). The spectrum of the PVDF initial material are marked as a continuous black line, while the other continuous lines stand for the PVDF coatings transferred by MAPLE and the dotted lines stand for the PVDF coatings transferred by MAPLE and those that were thermally-treated (MAPLE TT).

The FTIR vibrational bands of PVDF materials are summarized in Table 1; the chemical changes that occur in the PVDF samples deposited via the MAPLE technique at different laser fluences (1.0, 1.3, and 1.5 J/cm^2^) and those changes to the samples subjected to the thermal treatment (TT) procedure after deposition are shown in Figure 1.

In these spectra, the β phase—which is very important for the piezoelectric properties of PVDF material [50]—is evidenced particularly by the absorbance maxima at 840, 882, and 1276 cm^−1^ [50,51,52,53].

Nevertheless, the spectral lines for the initial bulk PVDF sample (black line in Figure 1), are characterized by the following chemical vibrations: the skeletal bending of -CF_2_ at 763 cm^−1^ [53] and the rocking vibration ρCH_2_ at 795 cm^−1^ peak [53]. The γ-phase of PVDF appears at 833 cm^−1^ [51], while the ρCH_2_ [52] rocking vibration was observed at 873 cm^−1^. Moreover, the twisting CH_2_ vibration and the stretching vibration of -CF_2_ of PVDF were identified at 973 cm^−1^ and 1070 cm^−1^, respectively [53]. Furthermore, at 1172 cm^−1^ the C-C [54] vibration from PVDF was observed. The spectral range between 1240 and 1400 cm^−1^ can be assigned to the long trans-sequence of the ferroelectric β-phase of PVDF [50,51,52,53] and ωCH_2_ [54] (Table 1).

Further, the analogous spectra obtained from pure PVDF material have been compared with those of MAPLE TT samples (dotted line spectra), and the small shift of the peak from 1276 cm^−1^ (MAPLE) to 1279 cm^−1^ peak (TT) characteristic to β-phase [51,52] was observed. The intensity of the bands at 840 and 882 cm^−1^, as well as at the 1276 cm^−1^ to 1279 cm^−1^ peaks [51,52,53], increases after MAPLE deposition and thermal treatment. It has also been found to be more intense at the laser fluence of 1.5 J/cm^2^ if compared with the pure PVDF peak [52]. Also, it was observed that when comparing to the PVDF bulk material, the bands belonging to the α phase at 612, 763, 795, and 976 cm^−1^ are markedly weakened and even disappear from the PVDF coatings transferred by MAPLE at fluences of 1.0, 1.3, and 1.5 J/cm^2^.

One of the explanations regarding the disappearance of the bands at 612, 763, 795, and 976 cm^−1^ would be that the transfer/deposition of PVDF material by the MAPLE technique promotes β phase crystallization (Table 1) [51,52]. This could be supported by the fact that the thermal treatment could be used to improve the crystalline structure, minimize the porosity, and ensure the elimination of the residual solvent (DMSO) out of the PVDF thin films [1]. It was also reported by Wan et al. that an additional annealing procedure can be used to increase the degree of crystallinity and further align the CF_2_ dipoles, leading to higher piezoelectric and pyroelectric properties than those of PVDF homopolymers [55]. The thermal treatment was used also, besides blending, to tailor the piezoelectric properties of the PVDF material [51,55].

No other significant modifications in the chemical structures were noticed after MAPLE deposition (straight line spectra) for any of the used fluences (1.0, 1.3, and 1.5 J/cm^2^), and the typical signatures corresponding to the PVDF material of the bond features corresponding to the main functional groups were kept after the deposition process for PVDF (see Figure 1).

As previously mentioned, MAPLE is a quasi-dry technique [34,35] when using low volatile solvents and high fluences, and especially when high fluences are used, the larger particles ejected would keep the solvent embedded in their volume after deposition on the substrate [34]. Therefore, the presence of the residual DMSO solvent within the deposited material was observed in the samples obtained by MAPLE with highest fluence, while the peaks in the range 1500–2000 cm^−1^ corresponding to the signature of the DMSO have decreased significantly after being subjected to the thermal treatment (Figure 1). Nevertheless, the FTIR attenuated total reflectance (ATR) spectra, compared with the MAPLE and MAPLE TT spectra, revealed that at higher laser fluences the polymer structure is preserved, and even tends to be similar as the initial material, without a loss of functionality and maintaining the typical signature bands (Figure 1).

### 2.2. Elemental Composition

**Figure 2:**
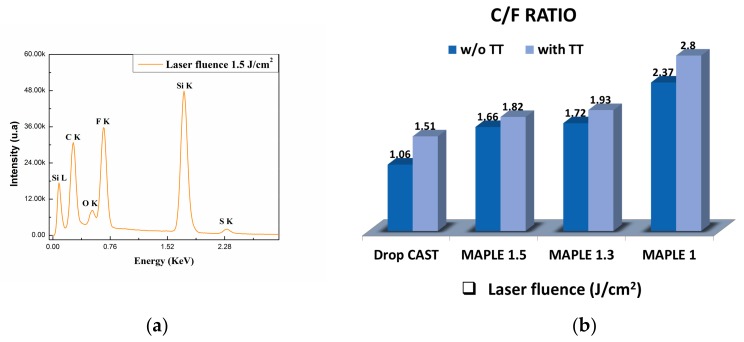
(**a**) Energy Dispersive X-Ray Spectroscopy (EDS) spectrum of PVDF thin film deposited by MAPLE at the highest laser fluence (1.5 J/cm^2^; see Figure 3e). (**b**) Graphic representation of the comparison of the C/F ratio in the PVDF coatings deposited by MAPLE and MAPLE TT at various laser fluences: 1.0, 1.3, and 1.5 J/cm^2^.

The elemental compositions of the deposited coatings obtained by MAPLE and also after the TT, unveiled carbon, fluorine, and oxygen as the main chemical elements originating from the PVDF structure, along with very low concentrations of sulphur (residue from DMSO). The data were obtained from three different regions within each surface, and showed that all of the coatings were uniformly formed on the substrate. A semi-quantitative relationship of the chemical elements can be calculated based on their peak sizes in the spectrum. Figure 2b shows changes in the ratios of the elemental components of coatings (C/F). It can be noticed that the TT on the PVDF films produces an increase in the C/F ratio if compared to the MAPLE-deposited films without TT. However, the increasing trend in the C/F ratio with decreases in the laser fluence may indicate that during thermal treatment, a large amount of carbon is incorporated into the surface of the coatings. All the aforementioned changes occur due to the fact that the C/F ratio undergoes some slight changes of the values, but the main functional groups of the polymer remain stable during laser irradiation, according to the FTIR analysis. These results show that the surface is uniform and the composition is preserved in the PVDF coatings when deposited at the highest fluence (1.5 J/cm^2^) and without TT (see Figure 1).

### 2.3. Surface Morphology

**Figure 3:**
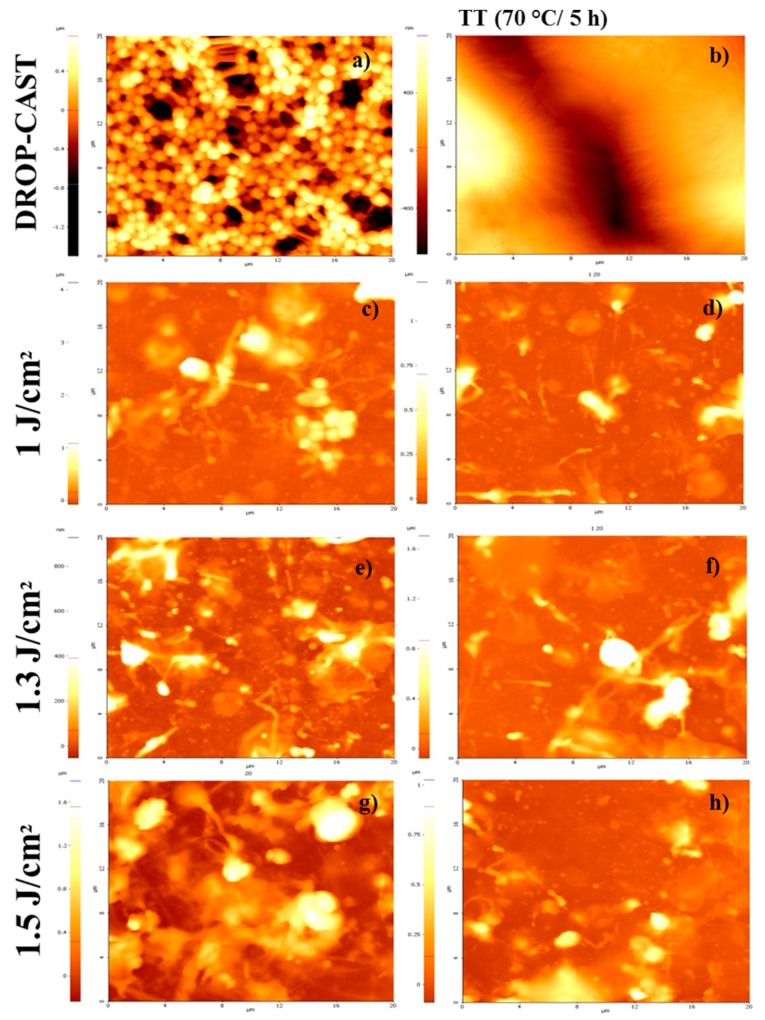
Atomic force microscopy (AFM) surface morphology images of PVDF thin films (20 × 20 μm^2^). Comparison between PVDF bulk, with (**a**) drop-cast; (**b**) drop-cast-TT, and the MAPLE-deposited samples at different fluences: (**c**) 1.0 J/cm^2^, (**e**) 1.3 J/cm^2^, (**g**) 1.5 J/cm^2^. In addition, the thermally-treated variant of the MAPLE-deposited films at (**d**) 1.0 J/cm^2^ + TT, (**f**) 1.3 J/cm^2^ + TT, and (**h**) 1.5 J/cm^2^ + TT.

The morphological characteristics of the PVDF coatings deposited by MAPLE with and without TT at different laser fluences (1, 1.3, and 1.5 J/cm^2^) were studied by atomic force microscopy (AFM) (Figure 3). The general observation is that the coatings obtained by MAPLE without TT are slightly smoother in terms or roughness, with the presence of several micro-sized particles or filaments with diameters ranging from 400 to 5000 nm, which were previously observed in other polymers for fluences above 0.8 J/cm^2^ [56]. According to the images presented in Figure 3a–e, the morphology of the control samples obtained by drop casting suffer significant modifications after applying the thermal treatment, with the morphology of the surface changing from globular-like structures to spherulite-like structures after treatment. However, in the case of MAPLE-obtained samples, the thermal treatment generates only slight modifications, especially related to elongation of the structures on the surface (Figure 3d,f,h).

The presence of filaments and island-like structures formed during MAPLE deposition can be explained based on some behavioural aspects: DMSO evaporates more slowly during deposition, and due to the highly hydrophobic nature of its polyvinylidene fluoride structure [12], there is a tendency to form island-like structures. Another explanation is related to the high laser fluence used, which has been shown previously to lead to filament-like structures. This type of rough and inhomogeneous globular morphology is consistent with previous reports of MAPLE films [1], originating from the mechanisms of target evaporation and ablation causing the polymer and solvent clusters to be ejected towards the substrate [56,57,58].

**Figure 4:**
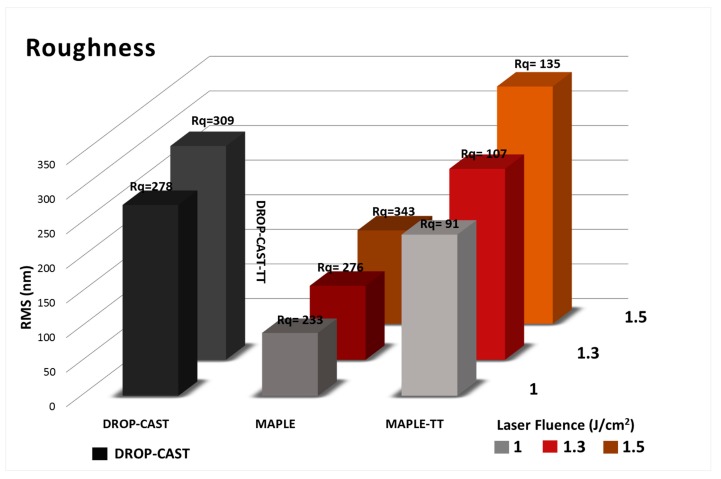
The roughness of the PVDF bulk: drop-cast and drop-cast TT, with thin films obtained by MAPLE deposition and subsequently thermally treated with different laser fluences of 1.0 J/cm^2^, 1.3 J/cm^2^, and 1.5 J/cm^2^ (from the AFM measurements on areas of 20 × 20 μm^2^ like in Figure 3).

If the MAPLE samples were characterized by the elongation of structures and the presence of micro-islands, some changes in surface morphology, including a decrease in the diameter in the case of TT samples, were observed, and this might be due to evaporation of the traces of solvent residue during TT. The elongation of the structures and the appearance of these micro-islands therefore determined an overall increase in roughness, as can be seen from Figure 4.

An important particularity of these surfaces of PVDF/MAPLE (Figure 3c,e,g) and PVDF/MAPLE TT (Figure 3d,f,h) is the micro-scale diameter increase with the laser fluence variation from 1.0 to 1.5 J/cm^2^, which had the effect of increasing the surface roughness from 91 ± 4 nm to 135 ± 7 nm. For further clarifications, the AFM images of the PVDF sample surface morphologies after TT are shown (Figure 3). The corresponding roughness values are reported in Figure 4 and represent the roughness of PVDF coatings measured on 20 × 20 μm^2^ area of deposited by MAPLE using different laser fluences. As mentioned above, the TT coatings have a higher roughness (Root Mean Square-RMS, increases approximately 2.5 times) if compared with the initial, thermally-untreated PVDF coatings (Figure 4), and the change in roughness value could be an indication that MAPLE and TT layers could be used for cells that prefer either smoother surfaces, such as fibroblasts, or those that tend to be favored by a roughness value of several hundred nm, such as osteoblasts.

### 2.4. Wettability Measurements

**Figure 5:**
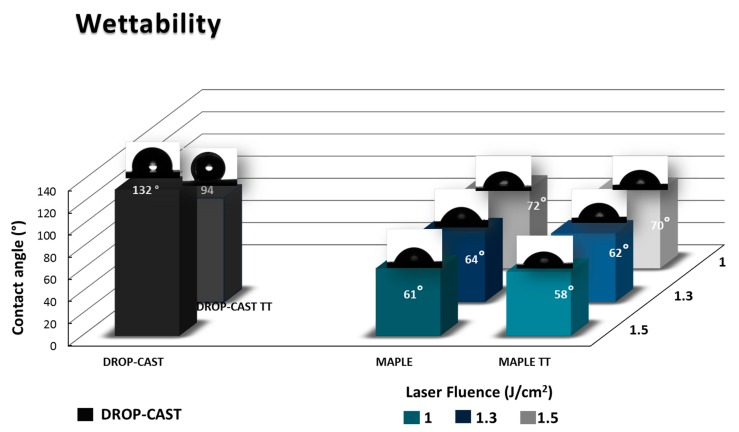
Contact angle comparison of PVDF bulk: drop-cast and drop-cast-TT with PVDF-MAPLE and PVDF-MAPLE TT at different laser fluences (1.0, 1.3, and 1.5 J/cm^2^).

Wettability represents a key factor when the biological interactions between cells or proteins with any material have to be considered. There are reports indicating that moderate hydrophilicity could conduct enhancement of the cells’ adhesion, survival, and biocompatibility. The comparative wettability of the different PVDF samples was determined by static contact angle measurements, as shown in Figure 5. In our case, the wettability measurements were assessed in order to determine how the laser fluence used before and after subjection to thermal treatment affects the surface characteristics of the PVDF layers deposited by the MAPLE technique, in comparison with those deposited by drop-cast (Figure 5).

Is well known that the PVDF bulk material has hydrophobic properties, which are due to the inclusion of fluorine atoms [12,13]; according to results in Figure 5, the values of the contact angles of various samples follow this sequence: drop-cast (132°) > PVDF, and drop-cast TT (94°) > PVDF-MAPLE (70°, 62°, 58°) > PVDF-MAPLE TT (72°, 64°, 61°) with increasing the laser fluence. Usually, in the case of polymers as PVDF-based fluoropolymers, which normally have hydrophobic properties [12], the decrease in the water contact angle and adjustment of the surface may be accomplished by introducing selected chemical compounds (by grafting, i.e., chemical bath deposition) or nanoparticles [25]. It is known that PVDF surfaces obtained by employing other deposition techniques like spin-coating or electrospinning are hydrophobic (90° and 120°, respectively) [12,16]. In our case, there are significant differences in the contact angles measured an all the samples obtained by MAPLE (values between 61° and 72°), as compared to the previously mentioned techniques; there were also differences from that measured on the drop-cast film (contact angle value of 132°). The changes to the contact angles could be correlated to the differences in surface physical and chemical characteristics when compared to drop-casted samples. If the drop-cast material presents globular particle-like shapes on the surface, with spherulite-like shapes after TT (Figure 3a,b), the change in material physical distribution onto the surface—as well as the slight increase in C/F ratio and the β-phase crystallization promotion, as observed earlier by FTIR (Table 1), which could further influence the alignment the CF_2_ dipoles [51,52,55] after MAPLE deposition and TT—could represent important factors in the change of surface contact angle values.

The fact that the surfaces obtained by MAPLE have contact angles with values corresponding to a hydrophilic character could represent one of the key factors involved in biological studies, suggesting that the need of any post-treatment to induce the hydrophilicity of PVDF coatings can be eliminated; however, further surface chemistry studies are required [12,51]. Nevertheless, overall, no significant wettability differences were observed between the MAPLE and MAPLE TT samples; the surface wettability decreases only by a small percentage, according to the modifications in the values of the roughness, which were discussed previously. Moreover, it is important to underline the stability of the TT PVDF layers when residual compounds should evaporate or a thermal sterilization of the medical assets would be requested.

### 2.5. In Vitro Preliminary Cell-Material Interaction

The compatibility between cells and synthetic polymers is strongly influenced by the biomaterial surface properties, such as the surface hydrophilicity, morphology [52], topography (roughness and thickness), surface energy, surface charge, and chemical composition [59,60]. As a consequence, the quality of cell-material interactions influences cell adhesion, migration, and proliferation, thus playing a critical role in the biocompatibility of scaffolds developed for tissue engineering purposes [61,62]. PVDF is reported to be a biocompatible thermoplastic with piezoelectric properties, which has been shown to provide advantageous properties as a scaffold for cellular attachment and growth [63,64,65,66]. In addition, due to the fact that bone has been proven to have piezoelectric behaviour [67], these types of materials are particularly appropriate for bone tissue engineering applications. In the present study, PVDF coatings deposited by the MAPLE technique demonstrated improved surface properties, including surface morphology, roughness, and wettability, all of which influence cellular responses. In this respect, it is crucial to evaluate the initial biological response induced by the newly developed PVDF layers by MAPLE deposition at various laser fluences, and also those that have been thermally treated (TT).

#### 2.5.1. Cell Attachment Behaviour

**Figure 6:**
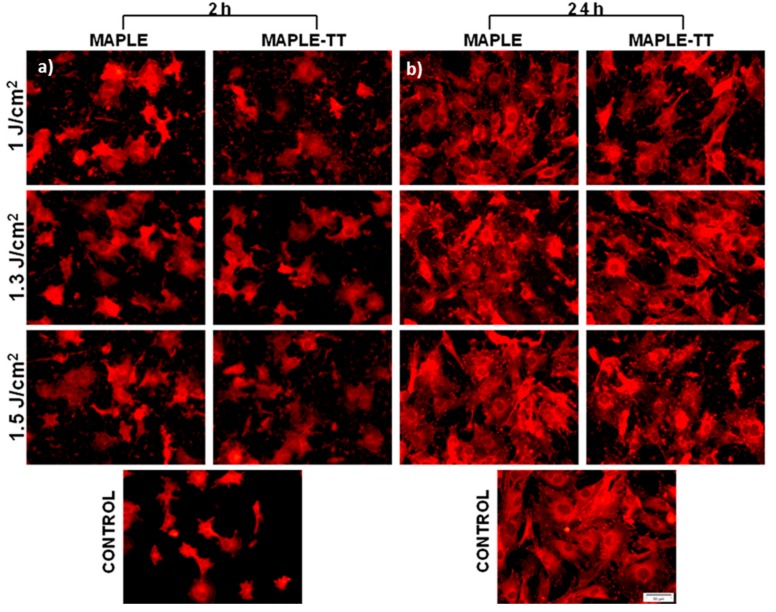
Immunofluorescence staining of vinculin (red) in MC3T3-E1 osteoblasts after 2 h (**a**) and 24 h (**b**) of culture onto different MAPLE and MAPLE TT-deposited PVDF materials. The controls were obtained on glass samples.

The initial adhesion of cells onto biomaterial surfaces is considered to be one of the most important steps in cell-material interaction [68]. As vinculin is an adaptor protein that acts as an adhesion molecule between cells and substratum [69], playing a key role in the formation of focal adhesions and also regulating cell adhesion [70], the capacity of MC3T3-E1 pre-osteoblasts to adhere to and proliferate onto each studied surface (Figure 6) is demonstrated by indirect immunofluorescence studies. The specific anti-vinculin antibody-marking experiments performed after 2 h of cell culture unveiled a homogenous repartition of endogenous vinculin into the cytoplasm of cells cultivated on all tested PVDF samples (Figure 6a). Regarding the cell morphology and cell area of the adhering osteoblasts, no significant differences were noted between PVDF films deposited at different fluences, as well as between TT and non-TT samples. Despite the presence of DMSO traces in the MAPLE coatings, no toxic effect on cells was observed. Cell spreading experiments at 24 h time point revealed a higher density of the adherent cells on all PVDF materials, as well as on the control substrate (Figure 6b). The fluorescent images show that the formation of vinculin-positive focal contacts increased progressively with cell culture time. Moreover, it was observed that after 24 h of culture, vinculin was mostly located at the periphery of cells at focal adhesion points. With respect to the shape of cells attached to the PVDF films, well-defined osteoblasts appear to be almost identical on all the tested substrates, and also similar to the control (glass surface). This study demonstrates that all candidate PVDF materials are able to support cell adherence, spreading, and the formation of focal contacts at 2 h post-seeding. Furthermore, these positive interactions between cells and material surfaces were maintained or even increased after 24 h of culture.

#### 2.5.2. Cell Viability

**Figure 7:**
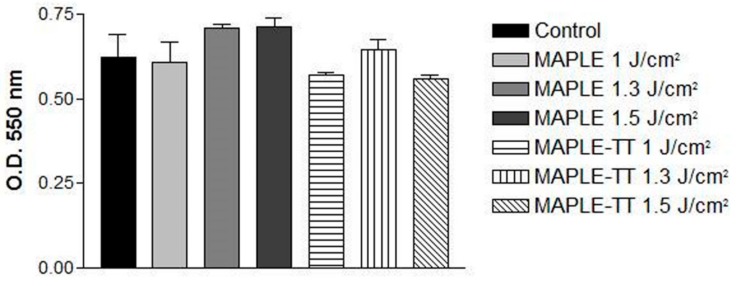
Cell viability of MC3T3-E1 cells assessed by MTT assay after 24 h of culture onto different MAPLE and MAPLE TT-deposited PVDF materials. Results are presented as mean ± standard deviation (SD) (*n* = 3).

The cell viability of MC3T3-E1 pre-osteoblasts on different MAPLE and MAPLE TT-deposited PVDF films was analyzed after 24 h of culture, using a 3-(4,5-Dimethylthiazol-2-yl)-2,5-diphenyltetrazolium bromide (MTT) test, which is shown in Figure 7. It was noticed that all surfaces supported the survival of viable osteoblasts with active metabolism, quite similar to the control glass sample.

Moreover, no significant differences in the optical densities between the studied PVDF samples and the control were noted. However, the TT applied to MAPLE deposited PVDF films seemed to slightly reduce the number of metabolically active cells at 24 h post-seeding. Overall, these results suggest that the novel PVDF substrates represent appropriate supports for promoting the adhesion and survival of MC3T3-E1 pre-osteoblasts within the first 24 h after seeding. It has been shown that surface characteristics, such as wettability and hydrophilicity [12], as well as roughness, have a deep influence on cellular behaviour, such as adhesion capacity; growth; and the morphological aspects of different cell types, including osteoblasts [71,72,73,74]. In our studies, the MAPLE and MAPLE TT-deposited PVDF thin films exhibit a hydrophilic character compared to the literature reference material, but no significant differences in wettability were remarked between the PVDF-based samples. Thus, the similar results obtained in in vitro cellular attachment and viability assays may be attributable to the similarities in surface properties.

## 3. Materials and Methods

### 3.1. Materials

Polyvinylidene fluoride (PVDF) was purchased from Sigma-Aldrich (427152 Aldrich, Saint Louis, MO, USA) in the form of beads, and was used without further purification. Target preparation PVDF beads were dissolved in dimethyl sulfoxide (DMSO) (276855 Aldrich, Saint Louis, MO, USA) solvent at a final 5 wt % concentration and at 70 °C, until a transparent and homogeneous solution was obtained. The solution was then frozen using liquid nitrogen, in a copper container that acted as a target holder, and was placed inside the deposition chamber. Double-polished n-type Si (001) plates for subsequent Fourier-transform infrared spectroscopy (FTIR) measurements and glass slides for cell culture studies were used as substrates. A standard cleaning procedure was applied to all substrates prior to their use. The substrates were carefully cleaned in ultrasonic baths, in acetone, ethanol, methanol, and deionized water, and blow-dried with nitrogen gas before usage.

### 3.2. Deposition Method: MAPLE Setup

The MAPLE (matrix-assisted pulsed laser evaporation) deposition [29,30,31] was carried out inside a vacuum chamber equipped with two vacuum pumps. The target was continuously cooled with liquid nitrogen throughout the deposition process. The frozen target was irradiated with a pulsed beam from a Nd:YAG laser (266 nm wavelength, 9 ns pulse duration, 10 Hz repetition rate), which was collimated by a convex lens and reached the target at a 45° incidence angle. During the deposition, the laser beam was translated using a scanning mirror, and the frozen target was rotated to allow uniform erosion of the target and to obtain a homogeneous deposition. The pressure inside the vacuum chamber was set at 10−4 mbar throughout the irradiation process. The polymer material was deposited on the substrates, placed 4 cm in front of the target and kept at ambient temperature. Each sample was deposited with 72,000 laser pulses. Before each the deposition, 3000 pulses were used to remove the surface layer of frozen water vapour formed on the target. The laser fluences were 1.0 J/cm^2^, 1.3 J/cm^2^, and 1.5 J/cm^2^.

### 3.3. Thermal Treatment (TT)

It was previously shown that MAPLE is a quasi-dry technique [34,35], and especially for high fluences and solvents with low evaporation rate, there are often cases of residual solvent present within the deposited material, which might affect its structure and functionality [34]. Therefore, the deposited coatings were subjected to a thermal treatment procedure (TT) at 70 °C for 5 h [49] to completely remove the residual DMSO solvent, according to the procedure by Mahdi [1].

### 3.4. Polyvinylidene Fluoride Coatings Characterization

#### 3.4.1. Thin Films Chemical Profile

The chemical structure of the PVDF thin films was determined by Fourier-transform infrared spectroscopy (FTIR) measurements. Characteristic vibrations of functional groups in the transferred material (MAPLE and MAPLE-TT) were compared to those of the initial material (PVDF beads). A Jasco FT/IR-6300 type spectrometer in the 400–7800 cm^−1^ range with a resolution of 4 cm^−1^ was used for the FTIR measurements. The spectra were measured in absorption mode, by the accumulation of 1024 scans, and in ATR (attenuated total reflectance) mode for the bulk of the PVDF (beads) for comparison. The elemental composition was evaluated quantitatively using a scanning electron microscope (FEI, model Inspect S) equipped with an energy dispersive X-ray spectrometer (Element Silicon Drift Detector). All data were acquired at an electron beam acceleration voltage of 5 kV and averaged from at least three different measurements.

#### 3.4.2. Morphological Characterization of the Deposited Polyvinylidene Fluoride Coatings

The surface morphology of the PVDF thin films was investigated by atomic force microscopy (AFM) on several areas with dimensions 20 µm × 20 µm, using a Park XE 100 AFM system with silicon tips in non-contact mode.

#### 3.4.3. Contact Angle Measurements

The contact angle measurements in static mode were obtained by a KSV CAM101 microscope. The sessile drop method was applied at room temperature using a syringe with double-distilled water, which delivered droplets with a volume of 1 μL. The reported values for the contact angle were obtained upon averaging five measurements performed on different areas of the sample.

### 3.5. In Vitro Biocompatibility Assessment

#### 3.5.1. Cell Culture

MC3T3-E1 pre-osteoblasts (American Type Culture Collection ®, CRL-2593TM) were cultured in basal medium Dulbecco’s Modified Eagle’s Medium (DMEM, Sigma-Aldrich Co., St. Louis, MO, USA), supplemented with 10% fetal bovine serum (FBS, Gibco, Grand Island, NY, USA) and 1% Penicillin/Streptomycin (10,000 units mL^−1^ penicillin and 10 mg mL^−1^ streptomycin) (Sigma-Aldrich Co., St. Louis, MO, USA) and incubated at 37 °C in a humidified atmosphere of 5% CO_2_. The cells were routinely expanded, and the medium was changed every 3 days. Prior to seeding the cells onto PVDF and glass substrates, these samples were sterilized by exposure to ultraviolet light for two hours (1 h on each side) and then placed in 24-well tissue culture polystyrene plates. For in vitro assays, MC3T3-E1 cells were seeded onto the samples at an initial density of 1 × 10^4^ cells/cm^2^ and allowed to adhere to the substrate for 15 min prior to filling the wells with 0.5 mL DMEM. Then, the cells were incubated in standard culture conditions for specific time points.

#### 3.5.2. Investigation of the Cellular Attachment

To investigate initial cell adhesion after 2 h of culture, as well as the cellular morphology of the MC3T3-E1 pre-osteoblasts after 24 h of maintenance in contact with the analysed substrates, an indirect immunofluorescence assay for the staining of vinculin was adopted. To highlight this cytoskeletal protein, known to be involved in cell adhesion and migration, the cells were washed twice with phosphate-buffered saline (PBS; Gibco, Grand Island, NY, USA), and were afterwards fixed with a cold solution of 4% paraformaldehyde (Sigma-Aldrich Co., Steinheim, Germany) in PBS. Further on, the cells were blocked and permeabilized with a solution containing 2% bovine serum albumin (BSA; Sigma-Aldrich Co., Steinheim, Germany) and 0.1% Triton X-100 (Sigma-Aldrich Co., Steinheim, Germany) in PBS. This step was followed by a 2 h incubation at room temperature, with a mouse monoclonal anti-vinculin antibody (Santa Cruz Biotechnology, Dallas, TX, USA), diluted 1:50 in a PBS solution containing 1.2% BSA. Finally, the samples were incubated for 30 minutes in the dark with a goat anti-mouse IgG secondary antibody coupled with AlexaFluor 546 (Invitrogen, Eugene, OR, USA), which was diluted 1:200 in PBS containing 1.2% BSA. Representative images were taken with an Olympus IX71 (Olympus IX71, Olympus, Tokyo, Japan) inverted fluorescence microscope and captured by means of Cell F image acquisition software (Version 5.0, Olympus Soft Imaging Solutions, Münster, Germany).

#### 3.5.3. Evaluation of Cell Viability

The mitochondrial activity of cells, which reflects the viable cell number, was measured by MTT (3-(4,5-Dimethylthiazol-2-yl)-2,5-diphenyltetrazolium bromide) (Sigma-Aldrich Co., Steinheim, Germany) assay. After 24 h of maintaining MC3T3-E1 cells in direct contact with the analysed samples, the culture medium was discarded and replaced with MTT solution (1 mg/mL) prepared in DMEM without serum. After 3 h of incubation at 37 °C, the supernatants were removed and the formed formazan crystals were solubilised in dimethyl sulfoxide (DMSO; Sigma-Aldrich Co., Steinheim, Germany); then the optical density was measured at 550 nm by means of an automatic microplate reader (Thermo Scientic Appliskan, Vantaa, Finland).

#### 3.5.4. Statistical Analysis

The quantitative data were obtained from triplicate samples and the results were expressed as mean ± SD (standard deviation). Statistical differences were determined by one-way ANOVA software (Bonfferoni’s multiple comparison test) using GraphPad Prism software (Version 3, GraphPad, San Diego, CA, USA); differences with *p* ≤ 0.05 were considered statistically significant.

## 4. Conclusions

In this work, the influence of MAPLE deposition parameters (laser fluence) and subsequently of thermal treatment (TT) on the physical, chemical, and morphological characteristics of the PVDF coating deposition, along with the effect on the in vitro pre-osteoblast response to surface characteristics were studied. From both FTIR and contact angle measurements, the results concluded that using MAPLE as deposition method not only allows for the successful transfer of PVDF as a coating, with preserved chemical structure as the initial bulk material, but also eliminates the need for any post-treatment modification of the PVDF film surfaces (a process that may sometimes be used, like the thermal treatment, to decrease the hydrophobicity of the surface). Still, the stability and the functional properties if subjected to a thermal sterilization process are preserved, indicating the process’ potential for other applications.

In vitro studies conducted with MC3T3-E1 pre-osteoblasts indicate that all surfaces supported the cellular survival and maintenance of an active metabolism, similar to the control sample. When TT was applied, a slightly smaller number of metabolically active cells at 24 h post-seeding were observed, but no significant differences in the optical densities between the analysed PVDF samples and the control were noted. Therefore, the novel PVDF layers can represent appropriate supports/substrates for promoting the adhesion and survival of MC3T3-E1 pre-osteoblasts. The overall results suggest that the novel PVDF coating materials could represent a promising perspective as candidates for bone tissue engineering applications. In order to emphasize the possible application of these films in bone tissue engineering, our further research will be focused on investigating the surface chemistry changes, by varying laser parameters and target compositions, as well as the ability of the new developed PVDF layers to sustain osteoblast differentiation.

## Figures and Tables

**Table 1:** Summary of the polyvinylidene fluoride (PVDF) coating bands before and after thermal treatment (TT), as well as the fingerprint of dimethyl sulfoxide (DMSO).

Characteristic Bands from PVDF (CH_2_CHF)_n_			
Peak Assignment of PVDF MAPLE		MAPLE TT	
1400 cm^−1^	ωCH_2_ wagging	1400 cm^−1^	ωCH_2_ wagging
1276 cm^−1^	Can be assigned to the long trans-sequence of the ferroelectric β-phase of PVDF	1279 cm^−1^	β phase
1068 & 1178 cm^−1^	νCF_2_ symmetrical stretching of -CF_2_ group; β-phase	1072 cm^−1^	CH_2_ wagging
		1178 cm^−1^	Symmetrical stretching of -CF_2_ group; β-phase
882 cm^−1^	C-F (stretching vibration); β phase	882 cm^−1^	β phase; C-F stretching vibration
879 cm^−1^	ν_sym_CC-C (asymmetric stretching vibration)	879 cm^−1^	ν_sym_CC-C (asymmetric stretching vibration)
840 cm^−1^	β-phase; C-F (stretching vibration); Deformation C-F from PVDF	840 cm^−1^	β-phase; C-F (stretching vibration); Deformation C-F from PVDF
Characteristic bands from DMSO (CH_3_)_2_SO			
1500–2000 cm^−1^		Fingerprint of DMSO	
